# Global patterns of conservation research importance in different countries of the world

**DOI:** 10.7717/peerj.2173

**Published:** 2016-07-05

**Authors:** Hideyuki Doi, Teruhiko Takahara

**Affiliations:** 1 Institute for Sustainable Sciences and Development, Hiroshima University, Higashi-Hiroshima, Japan; 2 Graduate School of Simulation Studies, University of Hyogo, Kobe, Japan; 3 Graduate School of Integrated Arts and Sciences, Hiroshima University, Higashi-Hiroshima, Japan; 4 Faculty of Life and Environmental Sciences, Shimane University, Matue, Japan

**Keywords:** Endangered species, Ecosystem management, Scientometric analysis, Biodiversity, Research activity, Publication, Conservation, Conservation biology

## Abstract

Conservation research is essential to help inform the science-based management of environments that support threatened and endangered wildlife; however, research effort is not necessarily uniform across countries globally. Here, we assessed how the research importance of conservation is distributed globally across different countries and what drives this variation. Specifically, we compared the number of conservation/ecological articles versus all scientific articles published for each country in relation to the number of endangered species, the protection status and number of ecosystems, and the economic status of each country (gross domestic product (GDP) per capita). We observed a significant and positive relationship between the proportion of conservation and ecology articles to all scientific articles with respect to the number of endangered species and the proportion of endangered species that are protected in a country, as well as GDP per capita. In conclusion, knowledge about the conservation and economic status of countries should be accounted for when predicting the research importance of conservation and ecology.

## Introduction

Much effort is undertaken to conserve biodiversity by ecologists, conservation managers, governments, and citizens (Primack, 1993; Margules & Pressey, 2000). Consequently, the scientific field of biological conservation is well established, with research over the last few decades focusing on how to conserve specific species or communities at ecosystem and global scales (Primack, 1993; Ratcliffe, 2012). However, the extent of research in any given area likely affects the conservation methods implemented and the actively used methods and conservation activity, both in a given country and at the global level. For example, the International Union for Conservation of Nature (IUCN) uses journal paper publications to assess the output of conservation studies globally, and used the results to plan conservation effort and add endangered species to the Red List (IUCN, 2013). Thus, we hypothesized that the importance and activity of conservation research is expected to induce conservation initiatives by the government and citizens.

The amount of funding and the political climate in a country represent potential factors that drive the level of conservation activity in different countries (e.g., Amano & Sutherland, 2013; McCreless et al., 2013; Lira-Noriega & Soberón, 2015). For example, conservation areas have higher management costs in politically stable countries, along with a greater numbers of citizen initiatives, such as nongovernmental organizations (NGOs) for conservation (McCreless et al., 2013). The national security of a country may also influence the level of biodiversity conservation activity (Amano & Sutherland, 2013). Success and impending opportunities in biodiversity policy and management are related to gaps in biodiversity monitoring (Pereira, Navarro & Martins, 2012). The status of biological conservation (e.g., protection activity and endangered species) may also be linked to the extent of conservation research activity in a given country. Thus, the global distribution of conservation research importance in different countries could help assess the global situation of biological conservation.

Three key factors may be related to the research importance of conservation in a given country: (1) biodiversity status, (2) funding and policy, and (3) economic status. The biodiversity status represents the number of endangered species and the area showing a decline in ecosystem functions and biodiversity, which may be associated with the level of conservation actions a given area (Pereira, Navarro & Martins, 2012; Archer et al., 2014). The funding and policies on conservation are important factors affecting conservation activity and expanding knowledge about target species (e.g., Kier et al., 2005; Archer et al., 2014; Martin et al., 2014). Finally, because developing countries tend to have smaller federal budgets, their level of research activity on conservation “hot topics,” such as the worldwide decline in pollinators, is much lower (Archer et al., 2014).

Scientific importance and activity have been estimated using a variety of parameters, such as the number of publications (Gu, 2004; Suluimanov, Frolova & Khasenova, 2009), the number of researchers, and research funding provided by the government (World Bank Group, 2013; Doi, Heeren & Maurage, 2014). Bibliometric analyses (e.g., assessing the number of publications) have been extensively used in scientometrics, including assessments on how the number of publications correlate with economic and biological factors, such as research funding and the traits of researchers (Grim, 2008). In addition, the number of publications has been used to analyze achievements within specific scientific fields (Doi, Heeren & Maurage, 2014; Nakano & Strayer, 2014). Such studies primarily focused on the economic/cultural state of countries; however, analyses investigating the importance of a specific field of research remain limited, particularly with respect to the fields of conservation and ecology.

Here, we aimed to estimate (1) the global distribution of conservation activity across all countries globally and (2) how this distribution correlates with the biodiversity status and socioeconomic factors within each country. We analyzed the global distribution of the research importance of conservation with respect to the proportion of conservation and ecological articles out of all scientific publications in a given country. In addition, we considered how the global distribution of the research importance of conservation correlates with the global dataset on biodiversity and its protection status, economic factors, and the geographical location of a country. Our results are expected to provide novel insight into the factors that drive the research importance of conservation and the repercussions on conservation activity in the field.

## Materials and Methods

### Collection of the publication data

For all countries, we collected all publications from journals categorized as “Nature and Landscape Conservation” in the SCImago database (SJR: SCImago Journal & Country Rank; retrieved on March 10, 2014; see http://www.scimagojr.com/countryrank.php for the dataset and details) from 1996 to 2012 (Falagas et al., 2008). We also collected all publications in journals categorized as “Ecology,” because we assumed that many ecological studies in the “Ecology” category address conservation biology. The data were obtained from the Scopus database (http://www.scopus.com). In the SCImago database, the journals were assigned to 313 specific subject categories based on Scopus classification. The country of origin for each paper was designated using the address of all of the coauthors listed in the Scopus database. This method ensured that all of the countries of the authors who collaborated on the paper were listed in the database. Our preliminary analysis showed a remarkable correlation between the number of conservation articles in countries and the number of the outstanding citations of the conservation articles (Pearson’s correlation coefficient *r* = 0.95; *p* < 0.001). Thus, we only used the number of published articles. We used the proportion of conservation or ecology articles of all scientific publications to normalize the research importance of conservation as a component of all scientific fields. We also determined the mean latitude of the country under study via Wikipedia.

### Collection of data on biodiversity and protection

We collected the following data on biodiversity and protection for all countries:

(1) We used the IUCN Red List database from 2008 to obtain the total number of endangered species in each country and region (http://www.iucnredlist.org, accessed on February 21, 2014). These species included reptiles, fishes, mollusks, and other invertebrates and plants (IUCN, 1994). We then calculated the percentage of endangered species that were presented in published papers versus all species documented in the 2008 Red List database for each country.
(2) We then calculated the percentage of the endangered species that protected according to the Convention on International Trade in Endangered Species (CITES) reporting requirements (CITES, 2000). Countries that have not ratified CITES were recorded as meeting 0% of the requirements. The data were compiled by the creators of the NationMaster website (http://www.nationmaster.com/country-info/stats/Environment/Endangered-species-protection, accessed on March 21, 2014). We used mean data from 1996 to 2010.
(3) We calculated the percentage terrestrial protected area using data from the World Bank Database’s website (http://data.worldbank.org, accessed on February 21, 2014). Terrestrial protected areas are completely or partially protected areas of at least 1,000 ha that are designated by national authorities as scientific reserves with limited public access, or are designated as national parks, natural monuments, nature reserves or wildlife sanctuaries, protected landscapes, or areas managed mainly for sustainable use. The data sources were obtained from the IUCN and UNEP-WCMC (IUCN, 1994; UNEP-WCMC, 2008). The indicator of protected-area coverage was calculated using all the nationally designated protected areas recorded in the World Database on Protected Areas (WDPA; http://www.protectedplanet.net). The terrestrial protected area was calculated using the WDPA as the percentage of protected terrestrial biomes weighted by national biomes (%). We used mean data from 1996 to 2012.

### Collection of data on economic and research activities

We collected data on economic and research expenditures for all countries. The following indicators of economic and research expenditure were retrieved from the website of the World Bank Database (http://data.worldbank.org, accessed on September 24, 2015):

(1) We calculated the gross domestic product (GDP) per capita from 1996 to 2012. GDP is the sum of the gross value added by all resident producers in an economy plus any product tariffs and minus any subsidies not included in the value of the products. This metric was calculated without adjustment for depreciation of fabricated assets and also did not account for the depletion and degradation of natural resources. GDP per capita was calculated as GDP divided by the midyear population size in each country.
(2) The percentage of GDP dedicated to research and development expenditure (hereafter, termed *research expenditure*) was calculated from the mean values for 1996 to 2010. Research and development covers basic research, applied research, and experimental development. Expenditure for research and development includes current and capital expenditures (both public and private) on creative work undertaken systematically to increase knowledge, including knowledge about humanity, culture, and society, and the use of this knowledge for new practical applications.
(3) We calculated the area covered by each country using data from 2010 from World Bank Database. This value represents total area of each country excluding the area covered by national claims to continental shelf, and exclusive economic zones. Inland water bodies (major rivers and lakes) were included in most cases.
(4) We calculated the mean percentage of forested areas in each country from 1996 to 2010. The percentage of forest cover in each country is the percentage of land used for natural or planted stands of trees of at least 5 m height, whether productive or not. However, the tree stands in agricultural production systems (e.g., fruit plantations and agroforestry systems) and trees in urban parks and gardens were excluded.
(5) We calculated the mean size of the human population of each country from 1996 to 2010. The total population of each country was based on the de facto definition of population, which counts all residents, regardless of legal status or citizenship. Refugees were excluded from the count, as they are not permanently settled in the country of asylum, and are generally considered part of the population of their country of origin.

### Statistical analysis

All statistical analyses were performed using R software, version 3.1.0 (R Development Core Team, 2013). We analyzed generalized linear models (GLMs) to estimate the relationships among the compiled factors and the proportion of conservation/ecology articles among all scientific publications. We used the Poisson error distribution for GLMs with a log link function prior to analysis, and the normality of each factor was verified using the Shapiro–Wilk normality test (*α* = 0.05). This test showed that all factors significantly deviated from the normal distribution. To account for deviation in the factors and variances, the factor values (except percentage data) were transformed using the log_10_(*x* + 1) equation for the analysis of GLMs. After transformation, we once again assessed the distribution using the Shapiro–Wilk normality test, and these transformed factors were not significant. The number of conservation and ecology articles was divided by the number of all scientific publications. Different countries had a different total number of publications; thus, we introduced an offset to GLMs, which is shown by the following equation:

\begin{eqnarray*}\text{Number of conservation or ecology articles}\nonumber\\\displaystyle \quad ={\lrm{\beta }}_{1}{\log \nolimits }_{10}\hspace*{1em}\text{Number of endangered species}\hspace*{1em}+\hspace*{1.99997pt}{\lrm{\beta }}_{2}\hspace*{1em}\text{Proportion of endangered species}\nonumber\\\displaystyle \qquad +\hspace*{1.99997pt}{\lrm{\beta }}_{3}\hspace*{1em}\text{Proportion of endangered}\hspace*{1.99997pt}\text{species under protection}\nonumber\\\displaystyle \qquad +\hspace*{1.99997pt}{\lrm{\beta }}_{4}{\log \nolimits }_{10}\hspace*{1em}\text{terrestrial protected area}\hspace*{1.99997pt}+\hspace*{1.99997pt}{\lrm{\beta }}_{5}{\log \nolimits }_{10}\hspace*{1em}\text{Country area}\nonumber\\\displaystyle \qquad +\hspace*{1.99997pt}{\lrm{\beta }}_{6}\hspace*{1em}\text{Percentage of forest area}\hspace*{1.99997pt}+\hspace*{1.99997pt}{\lrm{\beta }}_{7}\hspace*{1em}\text{Research expenditure}\nonumber\\\displaystyle \qquad +\hspace*{1.99997pt}{\lrm{\beta }}_{8}{\log \nolimits }_{10}\hspace*{1em}\text{GDP per capita}\hspace*{1.99997pt}+\hspace*{1.99997pt}{\lrm{\beta }}_{9}{\log \nolimits }_{10}\hspace*{1em}\text{Number of population}\nonumber\\\displaystyle \qquad +\hspace*{1.99997pt}{\lrm{\beta }}_{10}\hspace*{1em}\text{Mean latitude of country}\hspace*{1.99997pt}+\hspace*{1.99997pt}(\text{Intercept})+\text{offset [log (all publications)]} \end{eqnarray*}

where the coefficient of the offset was set to 1.0; the offset was on the log scale of the linear predictor according to the stated equation; and log was the function linking GLMs with the Poisson error distribution (Crawley, 2002). We selected the best models using downward stepwise methods with Akaike Information Criteria (AIC), calculated using the “PseudoR2” function in “BaylorEdPsych” ver. 0.5 package. The pseudo-*R*^2^ values of the models were calculated using the “PseudoR2” function method of McFadden and Nagelkerke.

\begin{eqnarray*}\text{McFadden:}\hspace*{1em}{R}^{2}=1-\ln \nolimits ({L}_{M})/\ln \nolimits ({L}_{0}) \end{eqnarray*}

where *L* represents the likelihood of the model. The rationale in the equation is that ln\nolimits (*L*_0_) is analogous to the residual sum of squares in the GLM. Thus, the *R*^2^ value from this formula corresponds to a proportional reduction in “error variance” and is used as a pseudo-*R*^2^ value for GLMs.

\begin{eqnarray*}\text{Nagelkerke:}\hspace*{1em}{R}^{2}=1-({L}_{R}/{L}_{F})^{2/N}/1-{L}_{R}^{2/N} \end{eqnarray*}

where *L* represents the likelihood of the model, while the *L*_*R*_ and *L*_*F*_ indicate the likelihood of intercept in the model and that of the specified model, respectively. *N* indicates the number of observations. Nagelkerke *R*^2^ adjusts the Cox-Snell *R*^2^ andthe range of values is |1|.

Also, we tested variance inflation factors (VIFs) for the four independent factors in the GLMs. The maximum VIF for all GLMs (maximum VIF = 1.77) was less than 2.0, indicating that the collinearity of the factors did not influence the GLM results. Pearson’s correlation coefficient was also used to test to what extent conservation and ecology publications were correlated. We set the significance level to 0.05 in all analyses.

**Figure 1:**
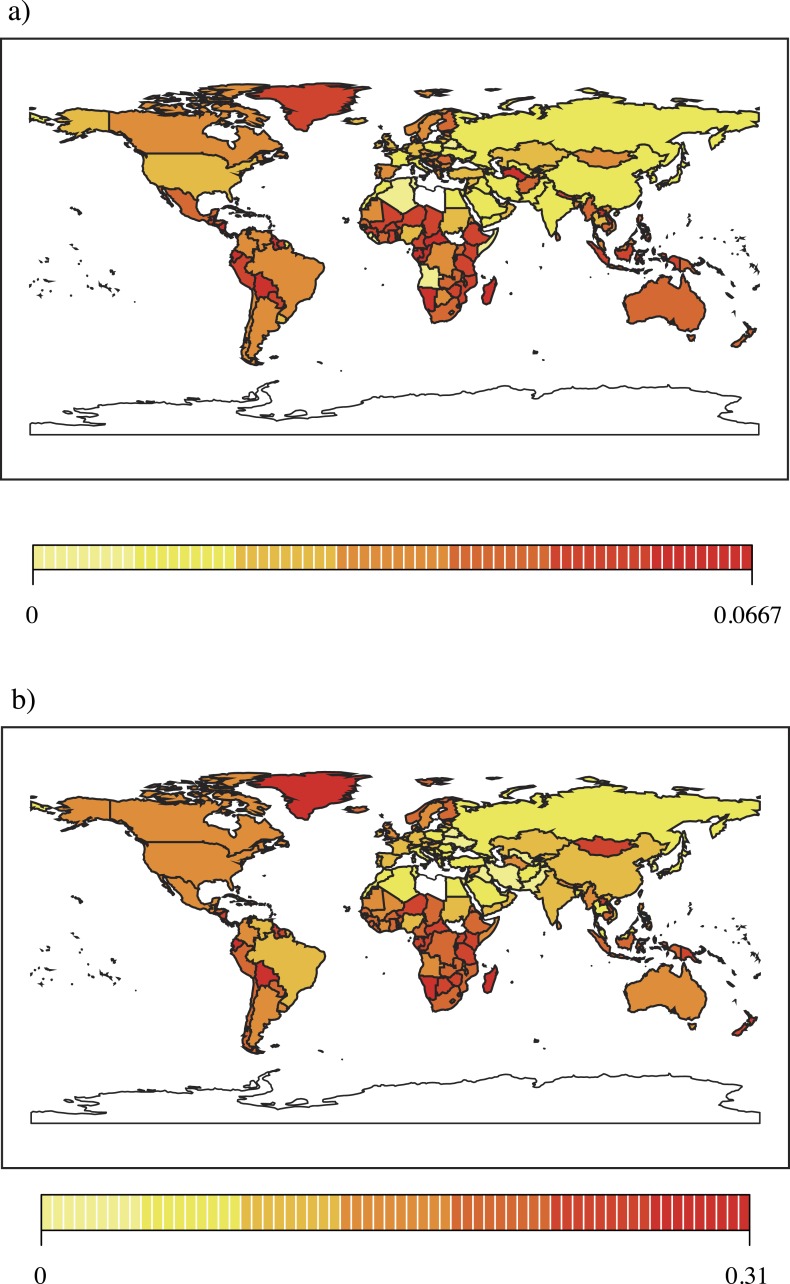
(A) Global distribution of the proportions of conservation articles among all scientific publications in various countries. (B) Global distribution of the proportions of ecology articles among all scientific publications in various countries. The colors denote the proportion according to the legend at the bottom.

## Results

The global distribution of the proportions of conservation and ecology articles among all publications varied among countries (Figs. 1A and 1B), with the proportion of conservation and ecology articles being particularly high in African and Asian countries. The global distribution of the proportion of conservation versus ecology articles was similar among all publications. The number of conservation articles was significantly correlated with the number of ecology articles (*r* = 0.89, *p* < 0.001: Pearson’s correlation coefficient).

**Figure 2:**
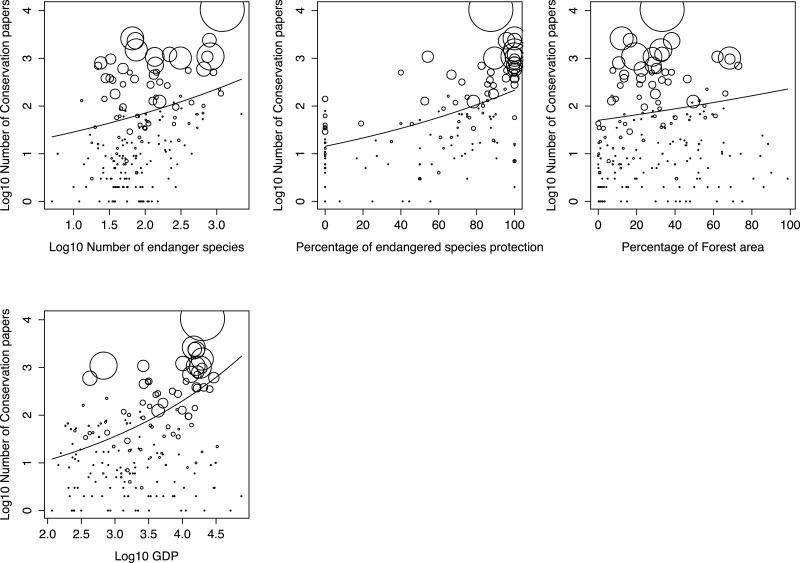
The relations between the proportion of conservation articles in a country and the four explanatory factors for the best generalized linear model (GLM). The bubble sizes indicate the number of all scientific publications in a country. The line indicates significant regression of the GLM.

**Figure 3:**
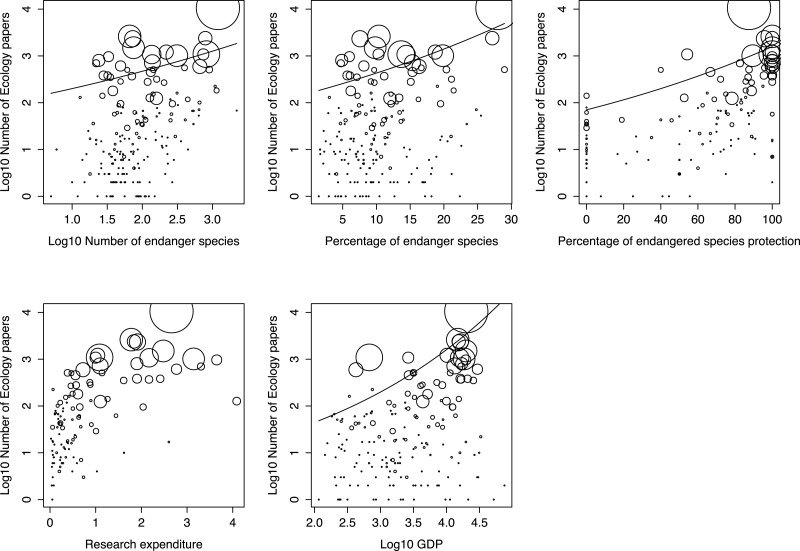
The relations between the proportion of ecology articles in a country and the five explanatory factors for the best generalized linear model (GLM). The bubble sizes indicate the number of all scientific publications in a country. The line indicates significant regression of the GLM.

The full and best GLMs for the proportion of conservation articles among all publications showed that the number of endangered species, the percentage of endangered species protection, percentage of forest area, and GDP were all significant factors for the best model. However, other factors, such as economic and scientific activity, were not included in the best model, despite being important for explaining these patterns (Table 1A). The full and best GLMs for ecological articles included the number of endangered species, percentage of endangered species, percentage of endangered species protection, research expenditure, and GDP. However, other factors, such as economic and scientific activity, were excluded (Table 1B). The number of endangered species, the percentage of endangered species protection, and GDP were significantly and positively correlated with the number of published conservation and ecology articles (Figs. 2 and 3, respectively). The correlations were also positive in countries with both very high and very low numbers of total publications.

**Table 1:** The results of analysis of generalized linear models (GLMs) for the proportion of (A) conservation and (B) ecology articles among all scientific publications.

	Full model				Best model			
	Coefficient	SE	*t* value	*p* value	Coefficient	SE	*t* value	*p* value
**(A) GLM for conservation articles**								
(Intercept)	−2.3981	**0.3754**	−6.387	** <0.001**	−2.231	**0.189**	−11.793	** <0.001**
Number of endangered species	–0.2160	0.2300	–0.939	0.3505	**0.176**	**0.046**	**3.862**	** <0.001**
Proportion of endangered species	0.2600	0.1597	1.628	0.1075				
Proportion of endangered species protection	**0.0020**	**0.0009**	**2.374**	**0.02**	**0.003**	**0.001**	**3.358**	**0.00117**
Terrestrial protected area	0.0324	0.1072	0.303	0.763				
Country area	–0.0045	0.0497	–0.091	0.9275				
Forest area	0.1373	0.0712	1.929	0.0573	**0.155**	**0.061**	**2.543**	**0.0128**
Research expenditure	0.0283	0.0357	0.792	0.4307				
GDP per capita	0.1348	0.0740	1.822	0.0723	**0.167**	**0.040**	**4.152**	** <0.001**
Number of Population	0.0429	0.0525	0.817	0.4166				
Mean latitude of country	0.0017	0.0034	0.509	0.6122				
Pseudo *R*^2^ (McFadden)				0.497				0.468
Pseudo *R*^2^ (Nagelkerke)				0.517				0.488
AIC				32.56				18.35
**(B) GLM for ecological articles**								
(Intercept)	–1.211	0.206	–5.883	** <0.001**	−1.179	**0.122**	−9.689	** <0.001**
Number of endangered species	**0.185**	**0.089**	**2.068**	**0.042**	**0.176**	**0.044**	**4.017**	** <0.001**
Proportion of endangered species	–0.193	0.129	–1.491	0.140	−0.215	**0.098**	−2.195	**0.031**
Proportion of endangered species protection	0.002	0.000	4.185	** <0.001**	**0.002**	**0.000**	**4.749**	** <0.001**
Terrestrial protected area	0.066	0.058	1.140	0.258				
Country area	0.036	0.027	1.305	0.196				
Forest area	–0.003	0.038	–0.068	0.946				
Research expenditure	0.029	0.020	1.461	0.148	0.031	0.019	1.669	0.099
GDP per capita	0.078	0.040	1.946	0.055	**0.099**	**0.034**	**2.915**	**0.005**
Number of population	–0.033	0.029	–1.127	0.263				
Mean latitude of country	0.001	0.002	0.642	0.523				
Pseudo *R*^2^ (McFadden)				0.555				0.536
Pseudo *R*^2^ (Nagelkerke)				0.565				0.546
AIC				28.54				16.34

**Notes.**

SE Standard error

To evaluate the explanation power for both GLMs, we calculated the McFadden and Nagelkerke pseudo-*R*^2^ values, which were around 0.5. These values indicated that the models explained 50% of variance in the proportion of conservation and ecological articles across all countries.

## Discussion

Here, we showed that a higher proportion of conservation and ecology articles among all scientific publications occur in the regions of Africa and Asia out of all regions globally. We found that factors on endangered species, including the number of endangered species and the proportion of endangered species under protection, were positively correlated with the proportion of conservation and ecology articles among all scientific publications in each country. Countries with a higher number of recorded endangered species and a higher proportion of protected endangered species appeared to have a greater proportion of conservation and ecology articles among all scientific publications. This finding is favorable for conservation causes, because countries that need more conservation initiatives for endangered species receive a higher level of importance in conservation research. Similar results were obtained for conservation and ecology articles among all publications. For example, including the four common factors in both of the best models indicated that ecological studies might also be performed in an area that needs conservation effort. This result was probably obtained because ecological studies provide baseline knowledge (e.g., traits and distributions of species) for people working on species conservation.

New knowledge may be gained by means of analyzing the research importance of conservation and the categorization of endangered species based on publications that describe the population size of target species. Thus, the research importance of conservation could be measured for new endangered species, along with the ecological traits and status of a species in a given country. A greater number of publications in the conservation field might increase the number of records of endangered species that fall in the Red List. Thus, such research importance in a conservation field could feed back into the categorization of endangered species (i.e., Gärdenfors et al., 2001). Research importance might be related to the state of conservation research importance in a country, such as through the proportion of the endangered species under protection. Thus, the positive correlation of the research importance of conservation with the proportion of the endangered species under protection might indicate that the research importance of conservation increases in a country that shows a higher level of protection activity for endangered species.

We also found that the proportion of forested area represented a significant factor for the best models. Of note, mostly natural forested areas were used in the analysis, not agricultural forests. The proportion of forested area might indicate the proportion of natural habitats in different countries, because forests generally support higher biodiversity and harbor more endangered species than other habitat types (Gentry, 1992; Lindenmayer & Franklin, 2002; Laurance et al., 2012), especially in tropical regions. This observation is particularly true for south Asian and west African countries, where we also observed a higher proportion of conservation and ecology articles (e.g., Gardner et al., 2009; Laurance et al., 2012). Thus, forested areas might represent an explanatory factor for the higher proportion of conservation articles in south Asian and west African countries.

Conversely, countries with few endangered species and a lower level of protection activity for endangered species have a lower importance level of conservation research. In such countries, scientific efforts and funds may be concentrated in other research fields. In fact, the proportion of publications in each scientific field differs among countries (Falagas et al., 2008).

Furthermore, the importance of conservation/ecological research increased with increasing GDP on average among different countries, as well as research expenditure (especially for ecological research). Thus, economical status and research funds may be important for increasing the number of publications in conservation/ecological fields. In fact, the economy of a country might represent an important determinant of the total number of scientific publications (Moed et al., 1985; Vinkler, 2008). In general, the number and quality of publications increase with research funding (Doi, Heeren & Maurage, 2014; Lin, Chen & Yang, 2014). The mean latitude of a country did not show any significant correlation with these factors. However, this type of geographical characteristic might not be related to the number of conservation and ecology articles published.

The global analyses of this study were performed using country-level datasets. Consequently, several limitations may exist. First, the personal level of research importance in the conservation and ecological fields are unknown. For instance, the personal level of research importance in conservation may be affected by the background of researchers, such as education, the standard of living, and experience with nature. Second, in our analysis, we used the mean values of the number of publications because the time series data on endangered species and their protection were not available. Yet, over the last two decades, there was a decline in endangered species populations, with new species being added to the Red List. In addition, the state of the economy and research expenditure of many countries has noticeably changed over time. Future studies using time series datasets should assess how long-term changes in research importance in conservation/ecology are linked with the long-term trends of the explanatory factors.

The results of this study could be applied to other research fields because the level of research importance in a scientific field could be related to social issues, such as climate change. This issue could be investigated in relation to global changes in biodiversity, the generation of energy for engineering projects, or changes to food production in agricultural fields. Furthermore, research importance may be linked to the scale of the measures undertaken against education on global issues (e.g., Bass et al., 2010; Lamanauskas, 2012). Thus, integrated research, involving both bibliometric and biological/political studies, of global datasets could prove interesting.

In conclusion, we showed the global distribution in the research importance of conservation and ecology as a proportion of all articles in the field of science. Statistical modeling of various potential driving factors (conservation, economic, and social states of the countries) showed that the conservation and economic states of countries are important when predicting the research importance of conservation/ecology in the field of science. We showed that the motivation for conservation increases the research importance of conservation and ecological studies.

##  Supplemental Information

10.7717/peerj.2173/supp-1 Table S1 The raw data for the analysis in this study

## Additional Information and Declaration

### Data Availability

The following information was supplied regarding data availability: The raw data has been supplied as Table S1.
